# Mitochondrial DNA mutations and male infertility

**DOI:** 10.4103/0971-6866.60183

**Published:** 2009

**Authors:** D. Prabhu Kumar, N. Sangeetha

**Affiliations:** Department of Chemical Engineering, Kongu Engineering College, Nanjanapuram, Erode - 638 107, Tamilnadu, India; 1 Department of Biochemistry, Kongu Arts and Science College, Nanjanapuram, Erode - 638 107, Tamilnadu, India

**Keywords:** Infertility, mitogenome, mitochondria, sperm motility

## Abstract

Infertility can be defined as difficulty in conceiving a child after 1 year of unprotected intercourse. Infertility can arise either because of the male factor or female factor or both. According to the current estimates, 15% of couples attempting their first pregnancy could not succeed. Infertility is either primary or secondary. Mitochondria have profound effect on all biochemical pathways, including the one that drivessperm motility. Sperm motility is heavily dependent on the ATP generated by oxidative phosphorylation in the mitochondrial sheath. In this review, the very positive role of mitochondrial genome's association with infertility is discussed

## Introduction

Approximately 15% of couples attempting their first pregnancy meet with failure. Data available over the past 20 years reveal that in approximately 30% of the cases, pathology is found in the man alone, and in another 20%, both the man and the woman are abnormal.[1] Therefore, the male factor is at least partly responsible in about 50% of infertile couples. In men, hormone disorders, illness, reproductive anatomy trauma and obstruction and sexual dysfunction can temporarily or permanently affect sperm and prevent conception. Some disorders become more difficult to treat the longer they persist without treatment.

The diagnosis of male infertility has been primarily based on the traditional semen analysis, as outlined by the World Health Organization guidelines, with a strong emphasis on the assessment of semen volume and sperm concentration, motility and morphology. About 75% of men who are infertile suffer from oligospermy, a very low sperm count or from asthenozoospermia, a condition in which many sperm are immotile. Both conditions are untreatable and poorly understood. A group of researchers in Spain has found genetic variants that correlate with a sperm's ability to swim.

Traditionally, genetic analysis has been based at the chromosomal level. Further, in order to explore the causes of unexplained infertility, there are several candidate genes that are being studied that could lead to future breakthroughs. Many studies indicated that the incidence of chromosomal abnormalities was correlated with the severity of male factor infertility but was mainly related to sperm concentration.[2] Multiple studies have been conducted to determine the incidence of Y-chromosome microdeletions. Other genetic causes involve CFTR mutations, *DAZL* gene mutation on autosomal chromosome 3 and many more. However, one area of genetic investigation, which has largely been ignored by reproductive biologists until recently, is the role of the mitochondrion and its genome. Mitochondria have a profound effect on all biochemical pathways, including the one that drives sperm motility.[3] Sperm motility is heavily dependent on the ATP generated by oxidative phosphorylation in the mitochondrial sheath. This review pays particular attention to the role of the mitochondrion and the mitochondrial genome (mtDNA) in male infertility.

### Mitochondrial DNA

Mitochondria are the intracellular organelles responsible for energy metabolism in eukaryotic cells. It was demonstrated more than 30 years ago that human and animal cells contain a second genome in the mitochondria.

### Molecular biology of mitochondrial genome

**Figure 1:**
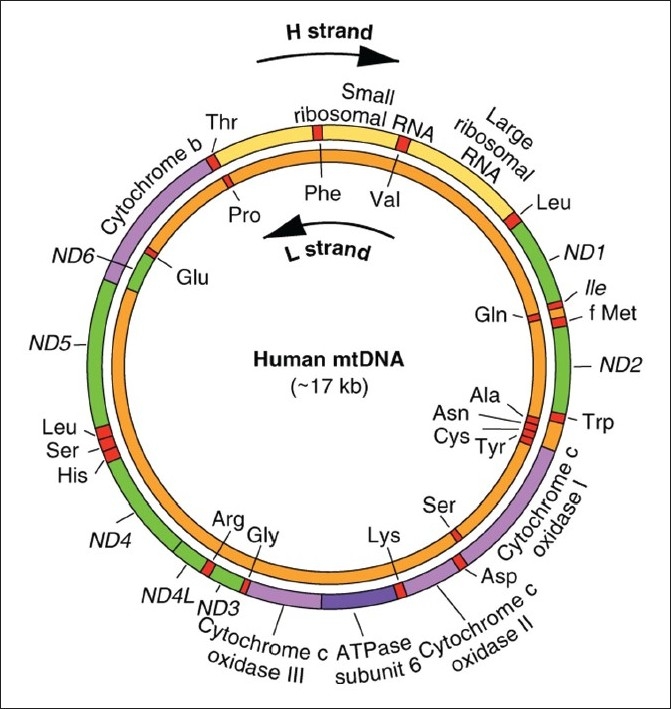
Human mitochondrial DNA

The important characteristics of human mtDNA are as follows:

- MtDNA is a small molecule, encoding a limited number of proteins and RNAs that are essential for the function of the mitochondrion
- MtDNA was first recorded by electron microscopy in 1963 and a year after it was isolated from Yeast mitochondria
- In 1981, the human mitochondria genome was completely sequenced and mapped with regard to gene context [4]
- The human mtDNA is a closed circular molecule, 16,569 bp in length, and is located within the mitochondrial matrix [Figure 1]
- It is double-stranded molecule containing 37 genes coding for 13 proteins, two rRNAs and 22 tRNAs, which are essential components of four respiratory enzyme complexes
- The 13 proteins are the components of the respiratory chain complexes. ND1, ND2, ND3, ND4, ND5 and ND6 are the subunits of complex 1. Cytochrome b is a subunit of complex 3. CO1, CO2 and CO3 are subunits of complex 4. ATPase 6 and 8 are subunits of complex 5. In addition, the nuclear DNA codes for the remaining subunits and those proteins that build or regulate the mitochondrion

### Features of mitochondrial genome

The major characteristic features of human mtDNA are as follows:

- mtDNA is maternally inherited.
- In each cell, there are 100-1,000 mitochondria depending on the type of cell, and there are two to 10 mtDNA per mitochondrion
- mtDNA is a semi-autonomous molecule that does not undergo recombination. It replicates rapidly without efficient proof-reading and DNA repair mechanisms
- A distinguishing feature is the almost total lack of intronic sequences in all genes, except one, i.e. the control region situated between tRNA-pro and tRNA-phe genes. Both strands are transcribed to synthesis of functional protein synthesis machinery in the mitochondria
- The peculiar structure and unique replication system of mtDNA and the highly oxidative environment in which it is located have caused it to mutate at a rate 10-20 times higher than that of nuclear DNA

In the past few years, more than 24 mutations of mtDNA have been identified and proven to be associated with human diseases.[5] Most of these mtDNA mutations are causally related to distinct neuromuscular and neurodegenerative diseases.[6] Because sperm movement requires a large amount of ATP to propel the flagellar apparatus, a defect in the mitochondrial respiratory function will cause a decline in motility and fertility.

**Figure 2:**
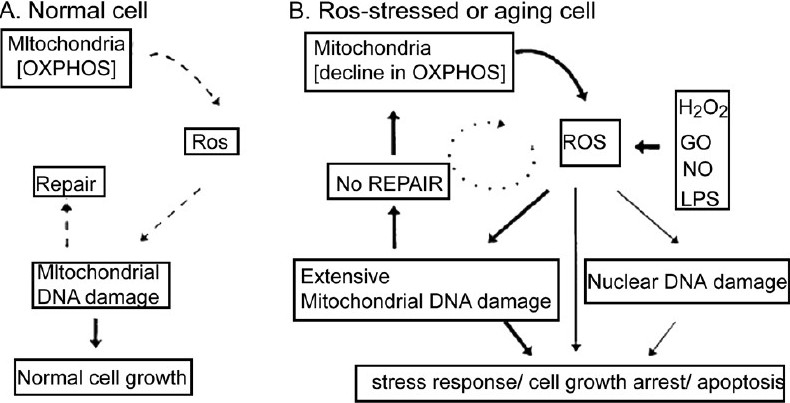
Schematic representation of the role of mitochondria in the generation of reactive oxygen species and oxidative stress

Studies have indicated that when compared with nuclear DNA, mtDNA contains an elevated basal level of base damage, such as 8-oxoguanine. The mitochondrion is responsible for oxidative phosphorylation, the biochemical pathway that generates ATP via the respiratory chain.[7] During this process, 1-2% of the oxygen that is consumed is released as reactive oxygen species (ROS), which can damage mtDNA and subsequently be repaired. However, under ROS-stressed conditions, the generation of ROS leads to persistent mtDNA damage [Figure 2]. The propagation of mitochondrial damage through the generation of secondary ROS could lead to a decline in oxidative phosphorylation and of mitochondrial physiology. In response to changes in mitochondrial physiology, stress response genes are activated and cells undergo growth arrest, which may be followed by apoptosis.[8]

### Mitochondrial respiratory chain

**Figure 3:**
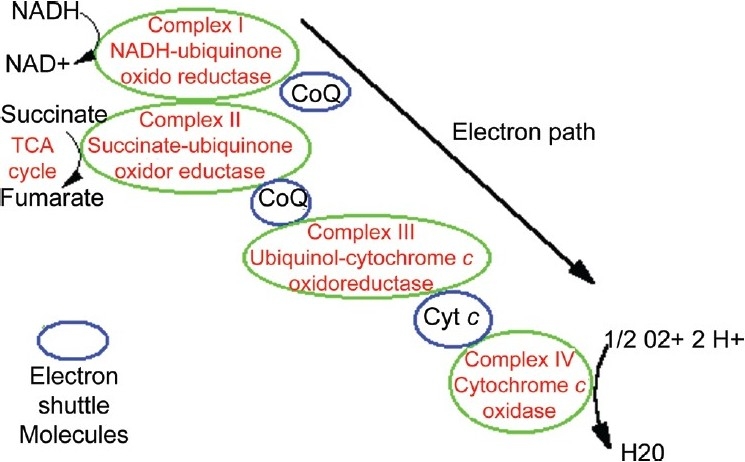
Mitochondrial respiratory chain

The respiratory chain is present in the inner mitochondrial membrane. It is composed of 80 peptides organized in five enzymatic complexes (I-V), electron shuttle molecules such as coenzyme Q (CoQ) and cytochrome c (Cyt c). The respiratory chain produces ATP by the reduction of oxygen to generate energy for cellular function. The pathway receives electrons generated by donors in intermediary metabolism. Electrons are sequentially transferred through redox groups to the final acceptor [Figure 3]. Oxygen-free energy generated is used to pump protons from the mitochondrial matrix to the intermembrane space. It generates an electrochemical gradient across the inner mitochondrial membrane. Protein flux back into the mitochondrial matrix through Complex V is coupled to ATP synthesis.

### Mitochondrial DNA mutations in human sperm

Sperms require a great deal of ATP for the flagellum to move around in the early phase of fertilization. It has been generally accepted that there are 70-80 mitochondria in the midpiece of mammalian sperm. It is long established that there is one copy of mtDNA in each mitochondrion in the midpiece of mammalian sperm.[9] Because the bioenergetic function of mitochondria is crucial for sperm motility, any quantitative or qualitative aberrations in mtDNA may affect the cellular functioning of spermatozoa.

Firstly, male infertility is associated with asthenozoospermia or oligoasthenozoospermia and has been reported in patients suffering from typical mtDNA diseases, involving point mutations or multiple deletions of mtDNA.[10] Secondly, sperms have been shown to be particularly prone to developing deletions of mtDNA. Some studies have shown that, in human sperm, these deletions are associated with a decline of motility and fertility. Thirdly, a correlation has been found between the quality of the semen and the functionality of the respiratory chain in sperm mitochondria. Moreover, it has been shown that mtDNA point mutations, mtDNA single nucleotide polymorphisms (SNPs) and mtDNA haplogroups can greatly influence semen quality.

The mtDNA molecules in human spermatozoa are vulnerable to oxidative damage and mutation and they play a role in male infertility.[2] About 85% of sperm samples contained large-scale mtDNA deletions of variable sizes and most spermatozoa had two to seven deletions of mtDNA. The genetic alterations in mitochondria could be pathological mutations or common mtDNA variants that only affect male fertility because mtDNA is maternally inherited. Thus, mutations responsible for encephalomyopathies decrease sperm motility and single or multiple mtDNA deletions have been associated with sperm dysfunction. Point mutations, deletions and the presence of a specific mtDNA haplogroup have been associated with poor sperm quality. Very low levels of somatic mtDNA deletions have been identified in the semen of infertile men. It has been suggested that these mutations cause infertility through an effect on sperm motility.

Among the mitochondrial deletions observed, the so-called “common deletion” of 4977 bp was the most prevalent and abundant one.[11] It remains to be investigated whether the mtDNA deletions are localized in the same mitochondria or in different organelles of spermatozoa harboring mutated mtDNA molecules. Some novel point mutations of mtDNA are present in some of the spermatozoa with poor motility or in spermatozoa of infertile males.

Ejaculated spermatozoa, particularly in infertile men, have been shown to display numerous features that are typical of apoptosis in somatic cells, including Fas expression, ROS production, activation of caspases, DNA fragmentation, reduction in mitochondrial membrane potential, plasma membrane translocation of phosphatidylserine and permeability. Deletion of sperm mitochondria-associated cysteine-rich protein, synonym mitochondrial capsule seleno protein, a structural protein associated with the keratinous capsules of sperm mitochondria, seriously affects sperm motility despite having normal sperm morphology. It has been shown that high levels of A3243G mtDNA mutant strongly correlate with low sperm motility.

### MITOMAP: A human mitochondrial genome database

MITOMAP is a comprehensive database of human mtDNA variation and its relationship with human evolution and disease.[12] MITOMAP attempts to integrate the broad spectrum of available molecular, genetic, functional and clinical information into a single unified entity that can be queried from a variety of different perspectives. The data content and operational tools of MITOMAP have been expanded over the past 5 years as the importance of mtDNA variation to human health has become increasingly apparent. The amount of curated mtDNA data has grown enormously in the past 6 years. Just in the past 2 years (September 2002-September 2004), the number of protein-coding gene mutations has increased 44%, the number of protein synthesis mutations has increased 58% and the number of somatic mutations has increased 52%.

### Mitoanalyzer

Mitoanalyzer is used to enable the investigator to determine the effects of any single base pair polymorphism or mutation in human mtDNA. The investigator enters the base pair number and whether the change is an insertion, deletion or substitution is identified by the mitoanalyzer. The program compares the sample sequence to the Cambridge Reference Sequence (CRS) and determines whether the single base pair change is a transition or transversion, whether it occurs in the non-coding (HV1 or HV2) or the coding region and whether it is in the first, second or third bp of the codon. Mitoanalyzer also provides information on whether the change affects a ribosomal RNA, a transfer RNA or a messenger RNA coding for a protein, whether it causes an amino acid change, the nature of that change, the position of the amino acid change in the protein and the new amino acid sequence of the changed protein as well as the original Cambridge Reference Sequence. Because a number of human diseases are known to be associated with specific mutations and deletions of mtDNA, mutation associated with published mitochondrial diseases are noted. Thus, this program facilitates rapid analysis and evaluation of SNPs and mutations found in human mtDNA.

## Conclusion

The important role of genetic abnormalities in the causation of human male infertility is increasingly recognized. While much remains to be learned in this fast-moving field, considerable progress has been made in the clinical delineation of genetic forms of male infertility and in the characterization of the responsible genes and their mutations or deletions. The mtDNA mutations detected so far may just represent the “tip of the iceberg” of all possible mutations in spermatozoa. Because sperms require a substantial amount of energy to swim fast enough to reach the oviduct during fertilization, the appropriate bioenergetic function of mitochondria is critical for male infertility.
